# PTEN loss promotes Warburg effect and prostate cancer cell growth by inducing FBP1 degradation

**DOI:** 10.3389/fonc.2022.911466

**Published:** 2022-09-27

**Authors:** Changze Song, Jianong Zhang, Xiao Liu, Meilu Li, Dejie Wang, Zhijian Kang, Jiaao Yu, Jiuwei Chen, Hongxin Pan, Honglei Wang, Guangbin Li, Haojie Huang

**Affiliations:** ^1^ Department of Urology, The Fourth Hospital of Harbin Medical University, Harbin Medical University, Harbin, China; ^2^ Department of Biochemistry and Molecular Biology, Mayo Clinic College of Medicine, Rochester, MN, United States; ^3^ Department of Urology, The Fourth Hospital of Harbin Medical University, Heilongjiang Key Laboratory of Scientific Research in Urology, Harbin, China; ^4^ National Health Commission (NHC) Key Laboratory of Molecular Probes and Targeted Diagnosis and Therapy, Harbin Medical University, Harbin, China; ^5^ Department of Dermatology, The Second Hospital of Harbin Medical University, Harbin Medical University, Harbin, China; ^6^ Department of Urological Surgery, Harbin Medical University Cancer Hospital, Harbin, China; ^7^ Department of Urology, Mayo Clinic College of Medicine, Rochester, MN, United States; ^8^ Mayo Clinic Comprehensive Cancer Center, Mayo Clinic College of Medicine, Rochester, MN, United States

**Keywords:** PTEN, SKP2, ubiquitination, PCa, FBP1

## Abstract

**Rationale:**

Fructose-1,6-bisphosphatase (FBP1) is a tumor suppressor and a key enzyme negatively regulating Warburg effect in cancer. However, regulation of FBP1 protein expression and its exact role in prostate cancer (PCa) is largely unclear. Phosphatase and tensin homolog (*PTEN*) is one of the most frequently deleted tumor suppressor genes in human PCa. However, the role of PTEN loss in aberrant Warburg effect in cancer remains poorly understood.

**Methods:**

Expression of PTEN and FBP1 was analyzed in several PCa cell lines and prostate tumor tissues in mice. Western blot (WB) and RT-PCR approaches were used to examine how PTEN regulates FBP1 expression. Co-immunoprecipitation (co-IP) and *in vivo* ubiquitination assays were used to define the regulatory mechanisms. A PCa xenograft model was employed to determine the impact of PTEN regulation of FBP1 on PCa growth *in vivo*.

**Result:**

We demonstrated that in a manner dependent of PI3K/AKT signal pathway PTEN regulated FBP1 expression in various PCa cell lines and tumors in mice. We confirmed that this regulation took place at the protein level and was mediated by SKP2 E3 ubiquitin ligase. Mechanistically, we showed that serine 271 phosphorylation of FBP1 by cyclin-dependent kinases (CDKs) was essential for SKP2-mediated degradation of FBP1 protein induced by PTEN loss. Most importantly, we further showed that loss of PTEN expression enhanced Warburg effect and PCa growth in mice in a manner dependent, at least partially on FBP1 protein degradation.

**Conclusions:**

Our results reveal a novel tumor-suppressive feature of PTEN in restraining FBP1 degradation and the Warburg effect. These results also suggest that prohibiting FBP1 protein degradation could be a viable therapeutic strategy for PTEN-deficient PCa.

## Introduction

Non-malignant cells primarily rely on mitochondrial oxidative phosphorylation to produce the energy needed in the processes of life. In contrast, one of the common characteristics of tumor cells is that they metabolize glucose into lactic acid even if there is sufficient oxygen, which is called aerobic glycolysis or “Warburg effect” (1). Indeed, mounting evidence suggests that this effect is closely associated with tumor carcinogenesis and progression (2). Furthermore, current research has suggested that this metabolic change can be accomplished by a variety of mechanisms (3), including the abnormal regulation of related enzymes in glucose metabolism (4).

Gluconeogenesis plays important roles in glucose homeostasis in normal cells. It is also essential in regulating aerobic glycolysis in cancerous cells (5, 6). Fructose-1,6-bisphosphatase (FBP1) is the rate-limiting enzyme in gluconeogenesis (7) and plays a key function in Warburg effect (4, 8–10). The loss of FBP1 may be an essential tumorigenic event that promotes the development of basal-like breast cancer cells in epithelial mesenchymal transformation (4). In gastric cancer, colon cancer and hepatocellular carcinoma, the expression of FBP1 is downregulated (8, 9, 11), and its deletion is related to the poor prognosis of clear cell renal cell carcinoma and hepatocellular carcinoma (10, 11). These studies have shown that FBP1 exerts a necessary function in regulating tumor glucose metabolism and cancer progression.

Prostate cancer (PCa) is the most commonly diagnosed malignancy and the second leading causes of death in American men (12). Despite the high morbidity and mortality of PCa, the understanding of molecular mechanisms related to the occurrence and evolution of this malignant disease is incomplete.

PTEN functions as a lipid phosphatase to dephosphorylate phosphatidylinositol (3–5) trisphosphate (PtdIns (3–5) P3 or PIP3) (13). It is a multi-functional tumor suppressor and is often lost in human cancer, including PCa (14–17). Elevated activation of the signaling networks through the phosphoinositide 3-kinase (PI3K) group of lipid kinases by loss of PTEN is a characteristic of most cancers such as PCa (18–21). Additionally, PTEN, as the main negative regulator of the PI3K/AKT pathway, is a key regulator controlling lipid and glucose metabolism and mitochondrial functions in cell (22). Previous studies have shown that the serine/threonine kinase AKT, as a downstream target of PI3K/PTEN, can phosphorylate a variety of metabolic related proteins, such as AS160, GSK3, FOXO and PGC-1a (23–26).

In this study, we found that PTEN inhibited the degradation of FBP1 protein, which was mediated *via* phosphorylation-dependent ubiquitination. Our results also suggest that the deregulation of FBP1 may be the key mechanism of tumor progression driven by Warburg effect in PTEN-deficient PCa.

## Materials and methods

### Cell lines and cell culture

22Rv1, DU145 and 293T cell lines were purchased from ATCC (Manassas, VA). PTEN WT and -null MEFs were originally generated from Pten knockout mice and kindly provided by Dr. Zhenbang Chen (Meharry Medical College, Nashville, TN). 22Rv1 and DU145 cells were cultured in RPMI 1640 medium (Corning Cellgro) with 10% fetal bovine serum (FBS) (Thermo Fisher Scientific). 293T cells and MEFs were cultured in Dulbecco’s modified Eagle’s medium (Corning Cellgro) supplied with 10% FBS. All cell lines were routinely cultured in 37°C, 5% CO_2_ incubator.

### Plasmids, antibodies, and reagents

The expression plasmids for Flag-FBP1, HA-FBP1, HA-Ub and HA-SKP2 had been generated in our lab. The Flag-FBP1 mutant S271A was generated by using the KOD-Plus Mutagenesis Kit (Toyobo). The antibodies used in this study include: FBP1 (Abcam); PTEN, AKT and pS473 AKT (Cell Signaling Technology); SKP2, ERK2 and p27Kip1 (Santa Cruz Biotechnology); Ser/Thr-p (BD Scientific); HA tag (Covance); β-Tubulin and Flag tag (Sigma). The chemicals used include: LY294002 (Invitrogen), MG132 (Millipore), CHX (Sigma) and Roscovitine (MedChemExpress).

### Western blot (WB)

The cells were harvested and lysed in RIPA buffer for more than 15 min on ice, and the lysate was centrifuged at 15,600 xg at 4°C for 10 min. After the supernatant was quantified using the BCA protein quantitative kit (Thermo Fisher Scientific), 4x DTT containing loading buffer (Thermo Fisher Scientific) was added to protein samples and the mixed samples were heated in 100°C for 5 min. The samples were separated on SDS-PAGE and transferred to nitrocellulose membrane (Thermo Fisher Scientific). The membrane was pre-blocked with 5% skim milk for 1 h at room temperature and incubated with primary antibody at 4°C overnight. Membranes were washed in 1x TBST for 3 times, 5 min each and then incubated with horseradish peroxidase-conjugated secondary antibody at room temperature for 1 h. Blots were finally visualized using SuperSignal West Pico Stable Peroxide Solution (Thermo Fisher Scientific).

### Reverse transcription and quantitative polymerase chain reaction (RT-qPCR)

Total RNA was isolated from cells by directly adding Trizol reagent (Thermo Fisher Scientific) into cultured cells. The first strand cDNA was synthesized from 1 µg of total RNA with GoScript kit (Promega). Real-time polymerase chain reaction (PCR) was carried by using SYBR green mix (Bio-Rad), C1000 Touch Thermal Cycler and CFX96 real-time system (Bio-Rad). All PCR signals were normalized to the internal control GAPDH cDNA, and the fold change was calculated using the 2^-△Ct^ method. The DNA sequence information of primers used for RT-qPCR is provided in **Table S1**.

### Co-immunoprecipitation (co-IP)

Co-IP was performed using the method reported previously (27). Cells transfected with expression plasmids were harvested and cells were lysed in IP buffer (Sigma-Aldrich, St. Louis, MO) on ice for 15 min or longer, and the cell lysate was centrifuged at 15,600 xg for 10 min at 4°C. The supernatant was transferred to a fresh tube. The supernatant was then incubated with protein A/G agarose beads (Thermo Fisher Scientific) and primary antibodies at 4°C overnight. The IP beads were washed extensively (six times) on ice with IP buffer, and then re-suspended in 1 × SDS loading buffer followed by SDS-PAGE and Western blot analysis.

### *In vivo* ubiquitination assays

293T cells were transfected with plasmids for HA–ubiquitin and related genes. After 36 h of transfection, the cells were treated with 30 μM of MG132 for 6 h, and then lysed in 1% SDS buffer and boiled for 10 min. The cell lysate was incubated with anti-FLAG M2 agarose beads (Sigma) at 4°C for 4 h. The beads was washed for 4 times using BC100 buffer containing 0.2% of Triton X-100. The pulldown proteins were eluted with 3X FLAG polypeptide at 4°C. Ubiquitinated proteins were analyzed by SDS-PAGE and Western blot.

### Colony formation assays

The procedure for colony formation assay was carried out as previously described (28, 29). In short, 1×10^3^ cells were plated into each well in 6-well plates. The next day, the cells were treated with vehicle or Roscovitine (10 μM, Cat# HY-30237, MedChemExpress). After about 16 days of treatment, the cells were fixed with 4% paraformaldehyde for 15 min, stained with (0.5% w/v) crystal violet for 1 h, and then gently rinsed with running water. The cell colonies with more than 50 cells were counted, and the survival curve was generated using linear regression.

### MTS assay

Cell viability was measured using an MTS kit (Promega) in accordance with the instruction from the manufacturer. Briefly, the cells were seeded into 96-well plates with about 1,000 cells per well. After the cells attached to the bottom of the well, the designated drugs were added to each well at the indicated concentration. At the specified time points, CellTiter 96R Aqueous One Solution Reagent with a final concentration of 10% (V/V) was added into the wells to measure cell viability. Absorbance was measured after incubating the cell culture plates in the cell incubator at 37°C for 60 min. The absorbance of 490 nm was read in a microplate reader.

### Gene silencing using small hairpin RNAs (shRNAs)

Lentivirus-based control and gene-specific shRNAs were purchased from Sigma-Aldrich. 293T cells were transfected using Lipofectamine 2000 with shRNA plasmids and virus package plasmids (PMD2.G and pSPAX2). Twelve hours after transfection, the spent medium was replaced with fresh DMEM containing 10% fetal bovine serum (FBS) and 1 mM sodium pyruvate. After 36 hours of transfection, the culture medium containing packaged virus was filtered using 0.45 μm filters and added to target cells by supplying 8 µg/mL polybrene. shRNA sequences are provided in **Table S2**.

### Measurement of glucose and lactic acid in cultured medium

Cells (4.0 × 10^5^/well) were seeded into each well of 6-well plates and cultured in phenol red-free RPMI 1640 medium (Thermo Fisher Scientific) for 24 h. The spent medium was collected and the glucose concentration of the spent medium was measured using the Glucose (GO) Assay Kit in accordance with the manufacturer’s instructions (Sigma-Aldrich). Glucose consumption was calculated based on the difference of glucose concentration between the spent and fresh medium. Lactate concentration of the spent medium was quantitatively measured using a L-Lactate Assay kit according to the manufacturer’s instructions (Eton Bioscience). Lactate production was calculated according to the difference of lactate concentration between the spent and fresh medium. The optical densities were measured at 570 nm wave-length in a plate reader.

### Generation of prostate-specific *Pten* knockout mice

All animal studies were approved by the Mayo Clinic Institutional Animal Care and Use Committee (IACUC). Mice were housed with a 12 h light/12 h dark cycle and free to eat and drink. Probasin (*Pb*)-driven Cre4 recombinase transgenic mice (C57BL/DBA2) were originally generated in the laboratory of Dr. Pradip Roy-Burnam at the University of Southern California, Los Angeles, CA (17) and acquired from the National Cancer Institute (NCI) Mouse Repository. *Pten* ^Loxp/Loxp^ conditioned knockout mice (129/Balb/c) were originally generated in the laboratory of Dr. Hong Wu at the University of California, Los Angeles, CA (18), and purchased from Jackson Laboratory (004597). Littermate mice were used for control and target mice to ensure comparable genetic backgrounds. The sequence information of all the PCR primers used is listed in **Table S1**.

### Generation of PCa xenografts in mice

SCID mice, at 6 weeks of age, were bred in house and randomly divided into different groups. The animal study was approved by the IACUC at Mayo Clinic. All mice were housed in standard conditions with a 12 h light/dark cycle and access to food and water ad libitum. DU145 cells (5×10^6^ in 100 μl 1×PBS) infected with lentivirus expressing empty vector (EV) or *PTEN*-specific shRNAs in combination with Flag-tagged FBP1-WT or S271A mutant were first mixed with Matrigel (BD Biosciences, 100 μl) on ice and then the mixed cells were injected s.c. into the right flank of mice. Xenograft growth was measured in a blinded fashion using a digital caliper and the volume of xenografts was estimated using the formula (L×W^2^)/2, where L is the length of tumor and W is the width. After the completion of measurement, mice were euthanized with CO_2_ and xenograft tumors were isolated and weighed. Tumor tissues were divided, and a portion was formalin-fixed and paraffin-embedded (FFPE) and the rest was frozen for protein and RNA extraction.

### Statistics

Unless otherwise specified, all experiments were repeated three or more times. One-sided or two-sided paired Student’s t test was used for single comparison, and multi comparisons were carried out using one-way ANOVA test. The P < 0.05 is considered statistically significant.

## Results

### PTEN positively regulates FBP1 in human PCa cell lines and murine prostate tumors

**Figure 1:**
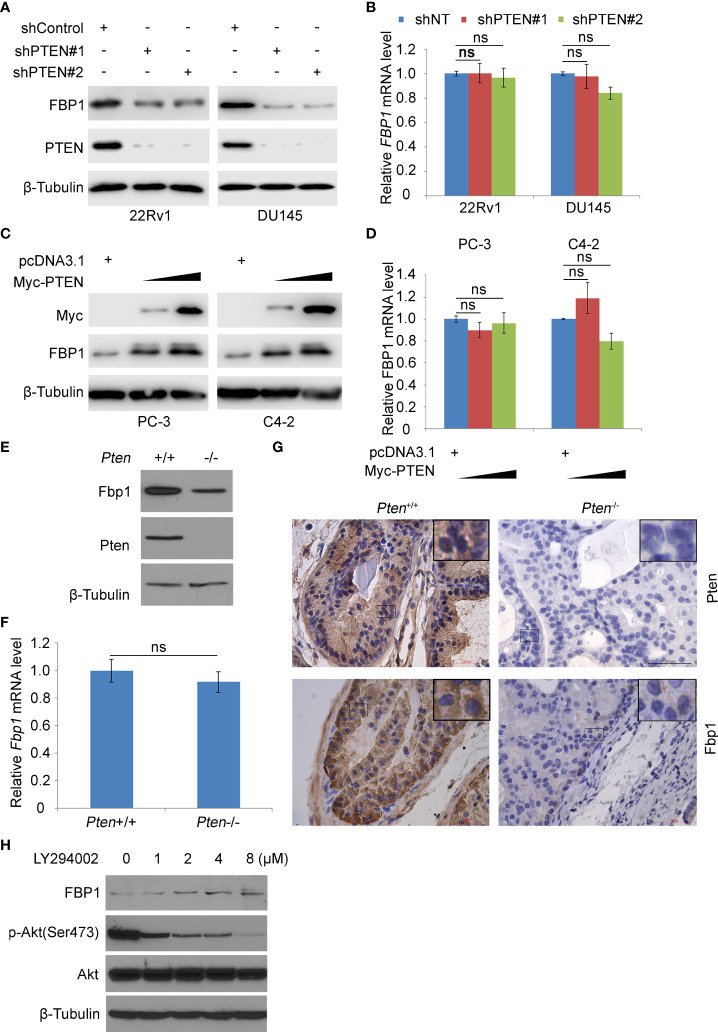
PTEN positively regulates FBP1 in human PCa cell lines and murine prostate tumors. **(A, B)** WB **(A)** and RT-PCR **(B)** were performed in 22Rv1 and DU145 cells infected with lentivirus expressing control or PTEN shRNAs for 48 h. β-Tubulin was used as a WB loading control. All quantitative data are shown as Mean ± SD (n=3). Student’s t test. n.s., not significant. **(C, D)** WB **(C)** and RT-PCR **(D)** were performed in PC-3 and C4-2 cells transfected with pcDNA3.1 or FBP1 expression plasmid for 24 h. All quantitative data are shown as Mean ± SD (n=3). Student’s t test. n.s., not significant. **(E, F)** WB **(E)** and RT-PCR **(F)** were performed in MEFs generated from *Pten* ^p/p^ conditional mice infected with or without infected with lentivirus expressing CMV-driven Cre. All quantitative data are shown as Mean ± SD (n=3). Student’s t test. n.s., not significant. **(G)** Photos of IHC of Fbp1 and Pten protein in the FFPE prostate tissues of Cre-negative mice (left column) and prostate tumors in Cre-positive mice (right column) at 5 months of age. **(H)** LNCaP cells were treated with LY294002 at the indicated concentrations for 24 h and then analyzed by WB.

Previous studies have shown that the loss of PTEN tumor suppressor leads to the abnormal activation of PI3K signaling pathway, which is considered to be one of the most common oncogenic events in the pathogenesis of PCa (21). FBP1 is a key rate-limiting enzyme in gluconeogenesis (30). Increasing evidence shows that FBP1 is a key enzyme in regulating tumor glucose metabolism, and the deletion of *FBP1* gene is related to cancer progression (4, 8–10). We were interested in investigating whether the expression of PTEN affected cancer metabolism by regulating the level of FBP1 in PCa cells. Therefore, we knocked down endogenous PTEN by the usage of two independent gene-specific small hairpin RNAs (shRNAs) in 22Rv1 and DU145 PCa cell lines. WB and RT-PCR showed that PTEN knockdown (KD) reduced the expression of FBP1 at the protein level, but had little impact on the level of *FBP1* mRNA (**Figures 1A, B**). Restoring PTEN in PTEN-negative PC-3 and C4-2 PCa cell lines induced FBP1 protein expression, but exerted little impact on *FBP1* mRNA expression (**Figures 1C, D**). Furthermore, we showed that deletion of *Pten* gene in MEFs induced downregulation of Fbp1 protein, but not *Fbp1* mRNA expression (**Figures 1E, F**). IHC analyses further showed that knockout of *Pten* gene in the murine prostate downregulated Fbp1 protein level in mice (**Figure 1G**). These results suggest that PTEN positively regulates FBP1 expression in different human PCa cell lines and mouse prostate tumors.

PTEN is an established negative regulator of PI3K/AKT signaling pathway (31). To determine how inhibition of this pathway affected FBP1 expression, PTEN-negative LNCaP PCa cells were treated with different doses of the PI3K inhibitor LY294002. We demonstrated that inhibition of AKT phosphorylation by LY294002 correlated with upregulation of FBP1 protein dose-dependently (**Figure 1H**). Collectively, these data suggest that PTEN regulates FBP1 protein expression *via* the PI3K/AKT signaling pathway.

### Loss of PTEN promotes FBP1 protein ubiquitination and degradation

**Figure 2:**
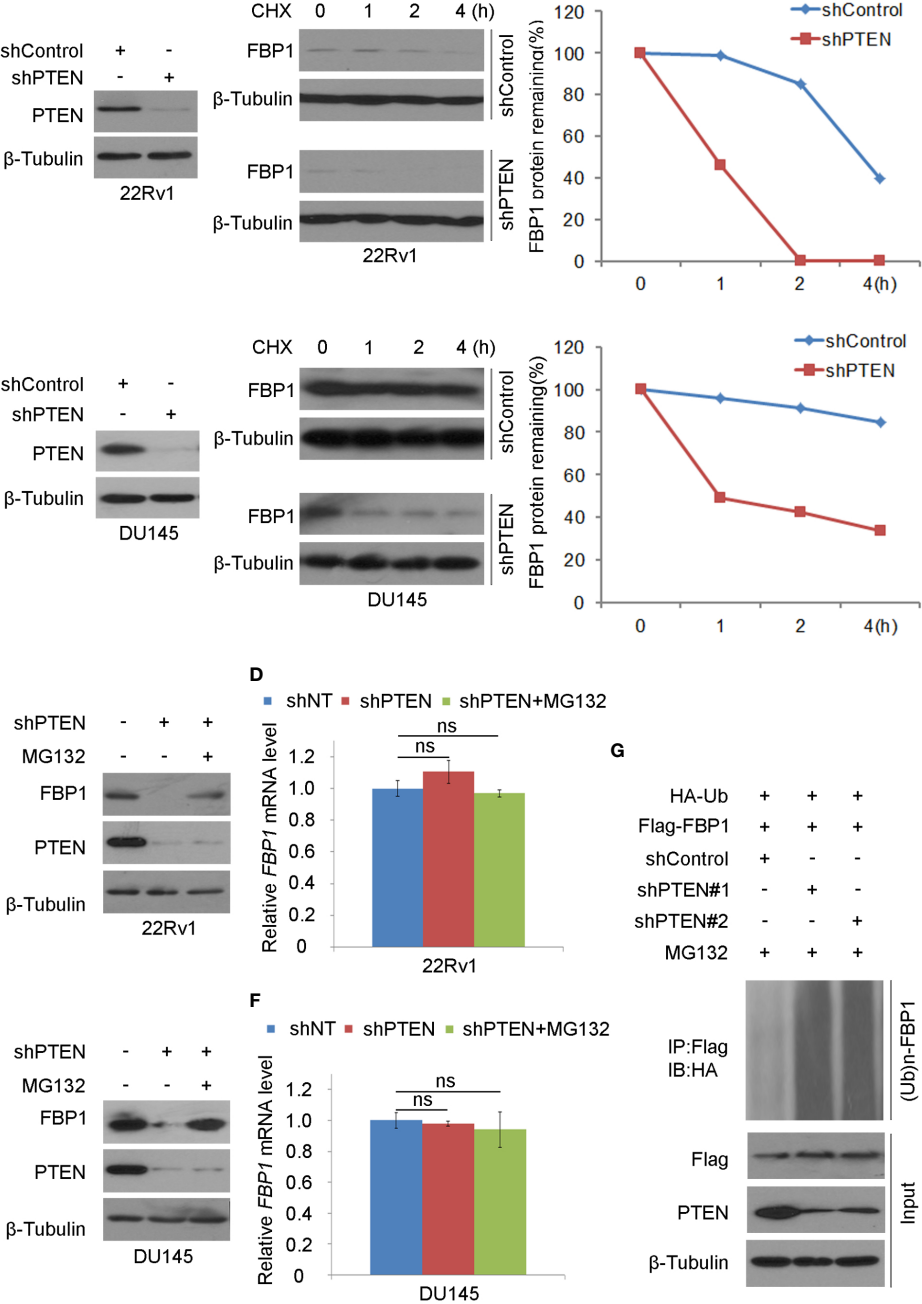
Loss of PTEN promotes FBP1 protein ubiquitination and degradation **(A, B)** WB and quantification of WB bands were carried out in 22Rv1 **(A)** and DU145 cells **(B)** infected with shControl or PTEN shRNAs for 48 h and followed by treatment with 50 μg/ml cycloheximide (CHX) for specific intervals of time. At every time point, the level of FBP1 protein was normalized to the level of β-Tubulin (a WB loading control) first and then to the value at the 0-h time point. **(C, D)** WB **(C)** and RT-PCR **(D)** were performed in 22Rv1 cells infected with lentivirus expressing control or PTEN shRNAs for 48 h and further treated with MG132 at 20 μM for 12 hours. All quantitative data are shown as Mean ± SD (n=3). Student’s t test. n.s., not significant. **(E, F)** WB **(E)** and RT-PCR **(F)** were conducted in DU145 cells infected with lentivirus expressing control or PTEN shRNAs for 48 h and exposed with MG132 at 20 μM for another 12 hours. All quantitative data are shown as Mean ± SD (n=3). Student’s t test. n.s., not significant. **(G)** WB evaluation was carried out using whole-cell lysate and co-IP samples obtained in 293T cells transfected with the indicated constructs and further treatment with 20 μM of MG132 for 12 more hours.

It has been reported that FBP1 can be degraded by ubiquitination (32). We sought to determine whether loss of PTEN regulated this process. To this end, we treated control and PTEN knockdown cells with the protein *de novo* synthesis inhibitor cycloheximide (CHX) and examined FBP1 protein expression level at different time points. We demonstrated that PTEN depletion largely decreased the half-life of FBP1 protein in 22Rv1 and DU145 cells (**Figures 2A, B**), suggesting that PTEN might negatively influence FBP1 protein degradation *via* proteasome pathway. To test this hypothesis, PTEN small hairpin RNAs (shPTENs) were transferred to 22Rv1 and DU145 cells and then treated with MG132. We found that PTEN loss-induced downregulation of FBP1 protein was blocked by MG132 treatment in both 22Rv1 and DU145 cells, but little or no effect was observed at the mRNA level (**Figures 2C–F**). In agreement with these results, the ubiquitin assays showed that PTEN depletion largely augmented FBP1 protein polyubiquitination (**Figure 2G**). Thus, these data suggest that loss of PTEN promotes the ubiquitination and proteasomal degradation of FBP1 protein in PCa cells.

### SKP2 mediates FBP1 protein ubiquitination and degradation induced by PTEN loss

**Figure 3:**
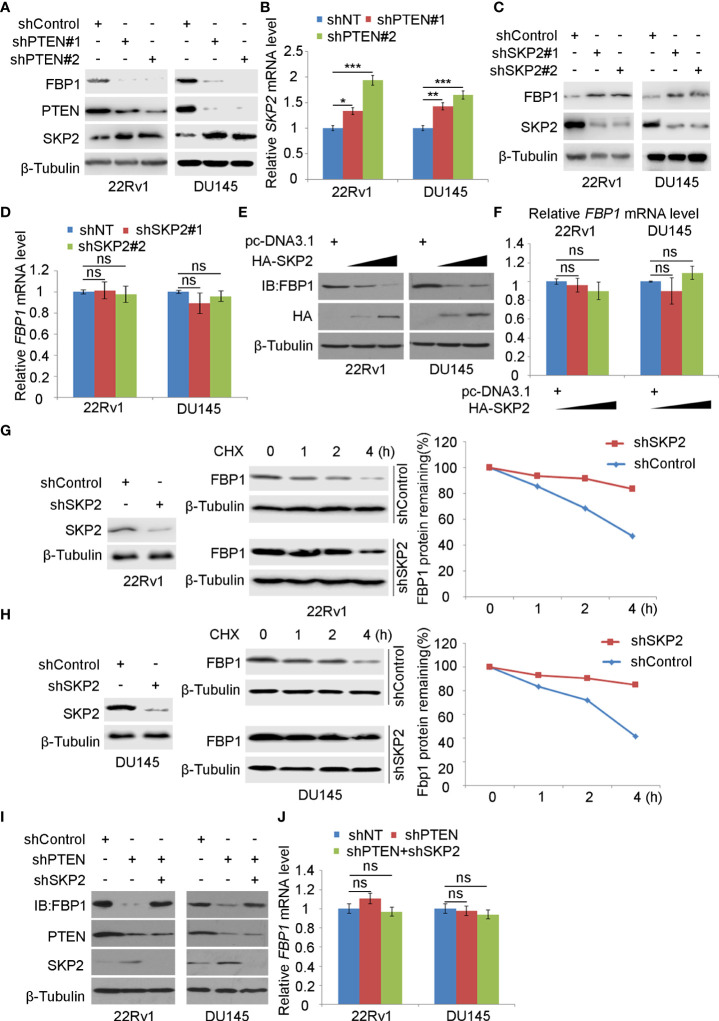
SKP2 mediates FBP1 protein ubiquitination and degradation induced by PTEN loss **(A, B)** WB **(A)** and quantitative RT-PCR **(B)** analyses were carried out in 22Rv1 and DU145 cells infected with lentivirus expressing control or PTEN-specific shRNAs for 48 hours. β-Tubulin was used as a WB loading control. All quantitative data are shown as Mean ± SD (n=3). Student’s t test. *P < 0.05, **P<0.01, ***P<0.001. **(C, D)** WB **(C)** and quantitative RT-PCR **(D)** analyses were performed in 22Rv1 and DU145 cells infected with lentivirus expressing control or SKP2-specific shRNAs for 48 hours. β-Tubulin was used as a WB loading control. All quantitative data are shown as Mean ± SD (n=3). Student’s t test. n.s., not significant. **(E, F)** WB **(E)** and quantitative RT-PCR **(F)** were conducted using samples obtained from 22Rv1 and DU145 cells transfected with pcDNA3.1 or HA-SKP2 expression plasmid for 24 hours. All quantitative data are shown as Mean ± SD (n=3). Student’s t test. n.s., not significant. **(G, H)** WB and quantification of WB bands were carried out in 22Rv1 **(G)** and DU145 cells **(H)** infected with shControl or SKP2-specific shRNAs for 48 hours and treated with 50 μg/ml cycloheximide (CHX) for different periods of time. At each time point, the intensity of FBP1 WB band was normalized to the intensity of β-Tubulin (a WB loading control) first and then to the value at the 0-h time point. **(I, J)** Western blot **(I)** and quantitative RT-PCR **(J)** analyses were conducted in 22Rv1 and DU145 cells infected with lentivirus expressing the indicated shRNAs for 48 hours. All quantitative data are shown as Mean ± SD (n=3). Student’s t test. n.s., not significant.

Consistent with the previous reports that inhibition of the PI3K/AKT signaling decreases expression of SKP2, an adaptor protein of the SKIP1-CULLIN1-F-Box protein (SCF) E3 ligase complex (33, 34), we found that depletion of PTEN increased mRNA and protein expression of SKP2 in 22Rv1 and DU145, which co-occurred with downregulation of FBP1 protein (**Figures 3A, B**). We, therefore, sought to determine whether SKP2 mediates FBP1 degradation induced by PTEN loss in PCa cells. To this end, shSKP2 was transfected into 22Rv1 and DU145 cells and protein expression was determined by WB. We showed that knockdown of SKP2 increased FBP1 expression at protein, but not mRNA level (**Figures 3C, D**). Moreover, we found that ectopic expression of SKP2 decreased FBP1 expression in a dose-dependent manner at the protein level, but had no drastic impact on *FBP1* mRNA expression (**Figures 3E, F**). Furthermore, we showed that knockdown of endogenous SKP2 caused stabilization of FBP1 protein in both 22Rv1 and DU145 cell lines (**Figures 3G, H**). Next, we sought to determine whether SKP2 mediates FBP1 degradation induced by PTEN loss. To this end, we knocked down PTEN and/or SKP2 in 22Rv1 and DU145 cells and we found that PTEN loss-induced downregulation of FBP1 protein was completed blocked by knocking down SKP2 in these two cell lines (**Figures 3I, J**). These data indicate that SKP2 mediates FBP1 degradation induced by PTEN loss in PCa cells.

### CDK inhibitor blocks FBP1 degradation induced by PTEN loss

**Figure 4:**
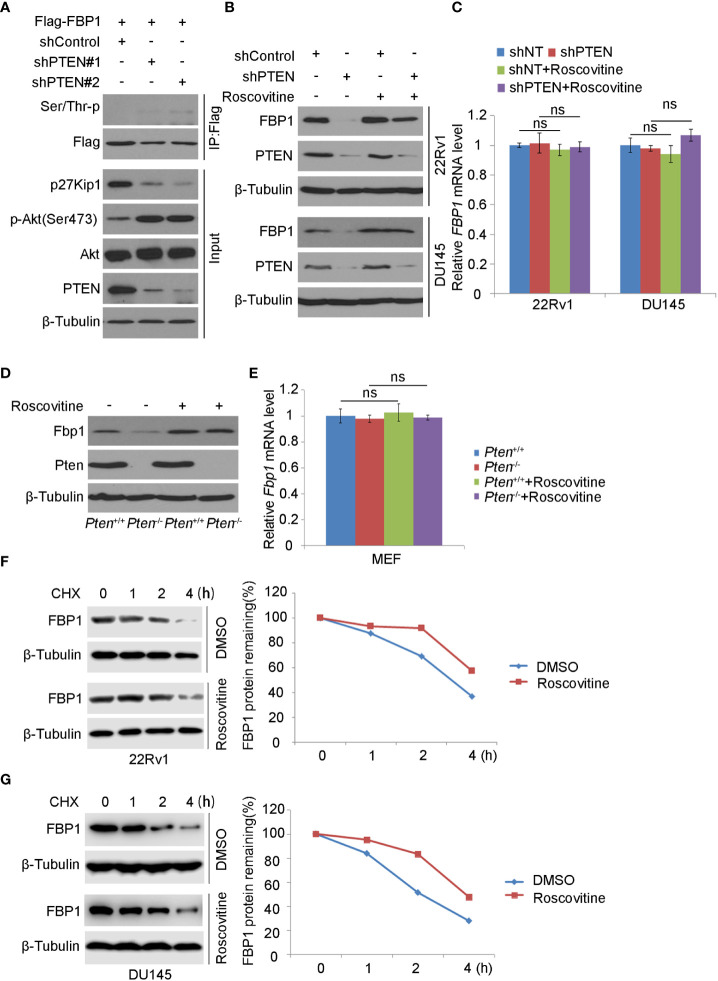
CDK inhibitor blocks FBP1 degradation induced by PTEN loss **(A)** WB was carried out using the whole-cell lysate and co-IP samples obtained from 293T cells transfected with the indicated constructs. β-Tubulin was used as a WB loading control. **(B, C)** Western blot **(B)** and quantitative RT-PCR **(C)** analysis in 22Rv1 and DU145 cells infected with lentivirus expressing control or PTEN-specific shRNAs for 48 hours and treated with Roscovitine for 24 hours. All quantitative data are shown as Mean ± SD (n=3). Student’s t test. n.s., not significant. **(D, E)** WB **(D)** and quantitative RT-PCR **(E)** were carried out in MEFs generated from *Pten* ^p/p^ conditional mice infected with or without lentivirus expression CMV-driven Cre and treated with Roscovitine for 24 hours. All quantitative data are shown as Mean ± SD (n=3). Student’s t test. n.s., not significant. **(F, G)** WB and quantification of WB bands were performed in 22Rv1 **(F)** and DU145 cells **(G)** treated with Roscovitine for 48 hours followed by treatment with 50 μg/ml cycloheximide (CHX) for different periods of time. At each time point, the intensity of FBP1 was normalized to the intensity of β-Tubulin (a WB loading control) first and then to the value at the 0-h time point.

It has been known that SKP2 induces K48-linked polyubiquitination and proteasomal degradation of p27Kip1 protein in a manner dependent on cyclin-dependent kinase-2 (CDK2)-mediated phosphorylation of p27Kip1 (35–40). Also, a study has shown that the deletion of PTEN leads to the activation of CDK2 by down-regulating p27Kip1 (41). As expected, we found that PTEN knockdown decreased p27Kip1 expression but most importantly, PTEN loss increased FBP1 phosphorylation (**Figure 4A**). To determine whether CDKs play any role in PTEN loss-induced downregulation of FBP1 protein, we knocked down PTEN in 22Rv1 and DU145 cells and treated cells with or without Roscovitine, a broad inhibitor of CDKs including CDK2 (**Figure 4B**). We demonstrated that Roscovitine treatment blocked the down-regulation of FBP1 protein caused by PTEN deletion in both cell lines, but without obvious impact on mRNA expression (**Figures 4B, C**). Similar results were obtained in wild-type (WT) and *Pten* knockout MEF cells (**Figures 4D, E**). We further showed that CDK inhibition diminished FBP1 protein decay in 22Rv1 and DU145 cells (**Figures 4F, G**). Together, these data indicate that CDKs mediate FBP1 degradation induced by PTEN loss and this process is blocked by CDK inhibitor.

### Serine 271 phosphorylation is important for FBP1 degradation induced by PTEN loss

**Figure 5:**
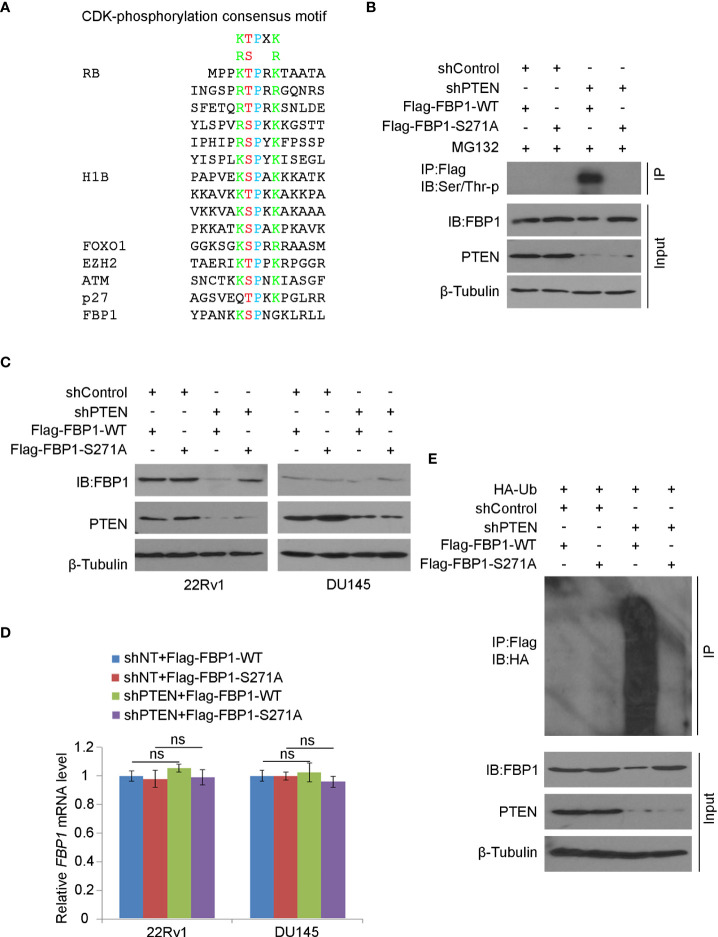
Serine 271 phosphorylation is important for FBP1 degradation induced by PTEN loss **(A)** Amino acid sequence alignment among CDK phosphorylation consensus motif, those identified in known CDK substrates, and the putative CDK phosphorylation site (serine 271) on FBP1. **(B)** WB analysis in the whole cell lysate and co-IP samples in 293T cells transfected with indicated plasmids or infected with indicated lentivirus for 48 hours. **(C, D)** WB **(C)** and quantitative RT-PCR **(D)** analyses were performed in 22Rv1 and DU145 cells infected with lentivirus expressing control or PTEN-specific shRNAs and/or transfected with plasmid for Flag-tagged FBP1 WT or S271A for 48 hours. All quantitative data are shown as Mean ± SD (n=3). Student’s t test. n.s., not significant. **(E)** WB analysis was carried out using the whole cell lysate and co-IP samples in 293T cells transfected with indicated plasmids or infected with indicated lentivirus for 48 hours.

It is known that CDKs regulate the protein level and function of their downstream substrates through protein phosphorylation (42). We noticed that akin to known CDK substrates such as RB, H1B, FOXO1, EZH2, ATM and p27Kip1, there is a putative CDK phosphorylation site (^270^KSPNG^274^) which is similar to the CDK phosphorylation consensus motif K/R-T/S-P-X-K/R (X, any amino acid) (**Figure 5A**). To determine whether the serine 271 (S271) residue plays any role in PTEN loss-induced FBP1 degradation, we generated an FBP1 S271A phosphorylation-resistant mutant. We demonstrated that knockdown of PTEN largely increased phosphorylation of Flag-FBP1-WT, but not the Flag-FBP1-S271A mutant in 293T cells (**Figure 5B**), suggesting that PTEN loss results in S271 phosphorylation on FBP1. We further showed that PTEN knockdown-induced downregulation of FBP1 was blocked by S271A mutation, but this mutant had no obvious effect on the mRNA expression of *FBP1* (**Figures 5C, D**). In agreement with these observations, *in vivo* ubiquitination assays showed that loss of PTEN robustly enhanced polyubiquitination of FBP1-WT, but not the FBP1-S271A phosphorylation-resistant mutant (**Figure 5E**). Thus, these data indicate that the serine 271 phosphorylation on FBP1 is necessary for FBP1 protein degradation induced by PTEN loss.

### FBP1 S271 phosphorylation is important in regulating Warburg effect and PCa growth

**Figure 6:**
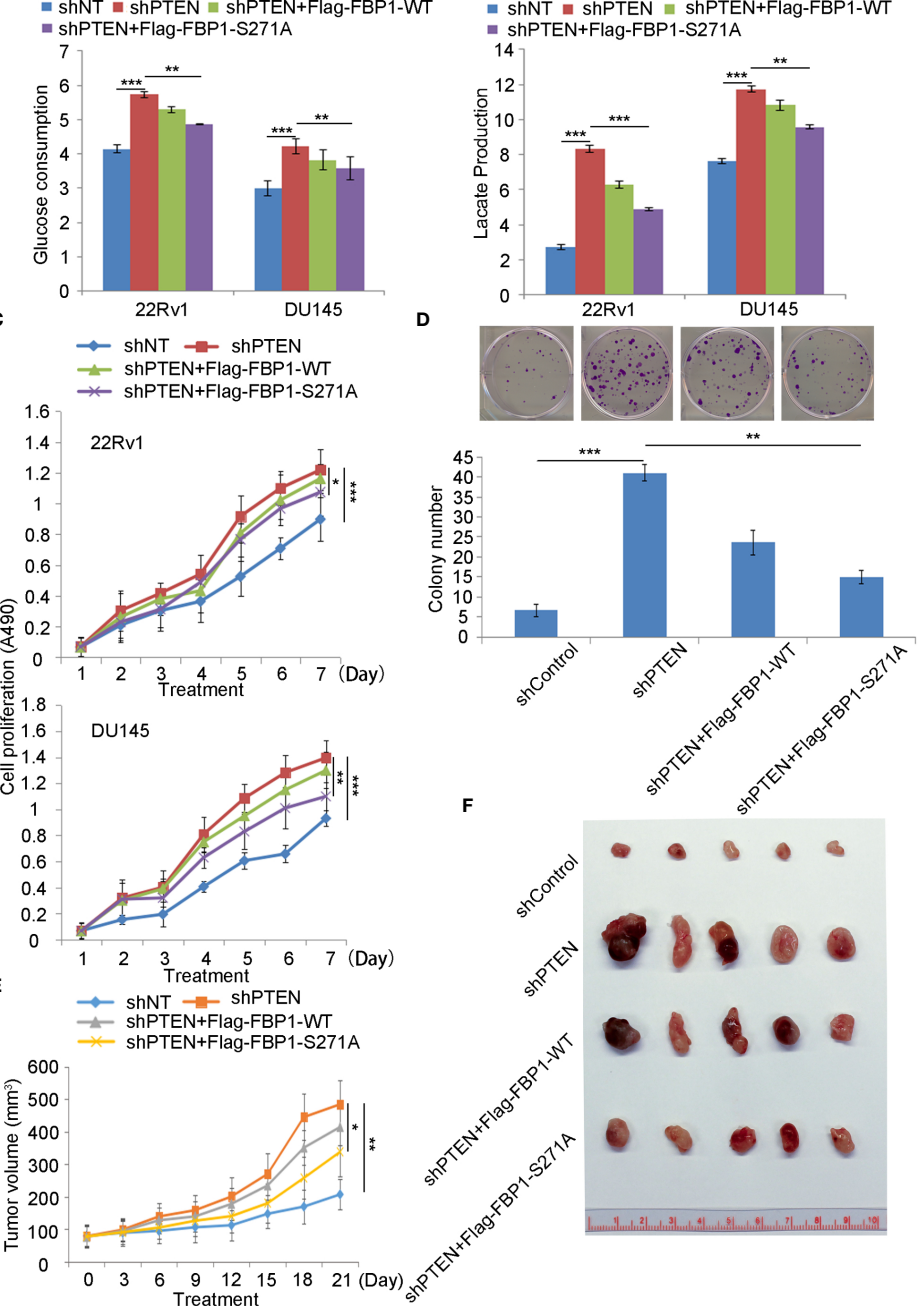
Serine 271 phosphorylation of FBP1 is important in regulating the Warburg effect and tumor growth in PCa **(A, B)** Glucose consumption and lactate production were measured in the spent medium of 22Rv1 and DU145 cells 48 hours after transfected with indicated constructs. All quantitative data are shown as Mean ± SD (n=3). Student’s t test. **P<0.01, ***P<0.001. **(C)** MTS assay was performed in 22Rv1 and DU145 cells 48 hours after transfected with indicated constructs for different periods of time. All quantitative data are shown as Mean ± SD (n=3). Student’s t test. *P<0.05, ***P<0.001. **(D)** Representative images were taken and quantification of the results of colony formation assays was performed in DU145 cells transfected with indicated constructs for 13 days. All quantitative data are shown as Mean ± SD (n=3). Student’s t test. **P<0.01, ***P<0.001. **(E, F)** Tumor growth curve over the time **(E)** and photograph **(F)** of tumors at the endpoint of inoculation of DU145 cells infected with lentivirus expressing indicated shRNAs or expression vectors. All quantitative data are shown as Mean ± SD (n=3). Student’s t test. **P<0.01.

Given that FBP1 is a major negative regulator of glycolysis, and cancer cells rely heavily on aerobic glycolysis (Warburg effect) for growth, we were very interested to determine whether restored expression of FBP1 inhibited the growth of PCa cells by blocking Warburg effect. As expected, we found that knockdown of PTEN increased glucose metabolism and lactate production in both 22Rv1 and DU145 cells (**Figures 6A, B**). This impact was largely inhibited by ectopic expression of the phosphorylation/degradation-resistant mutant Flag-tagged FBP1-S271A, but only modestly diminished by ectopic expression of Flag-tagged degradable FBP1-WT (**Figures 6A, B**). Similar results were seen in the cell proliferation assays in the two cell lines (**Figures 6C, D**). Next, we evaluated the impact of restored expression of FBP1 on tumor growth in mice. We infected DU145 cells with lentivirus expressing shControl or shPTEN in combination with or without Flag-tagged FBP1-WT or FBP1-S271A and inoculated these groups of cells into SCID mice. We discovered that FBP1-S271A expression, but not FBP1-WT inhibited PTEN knockdown-enhanced tumor growth *in vivo* (**Figures 6E, F**). Therefore, our data suggest that degradation of FBP1 plays a significant role in contributing to PTEN deletion-induced PCa growth *in vitro* and *in vivo*.

## Discussion

**Figure 7:**
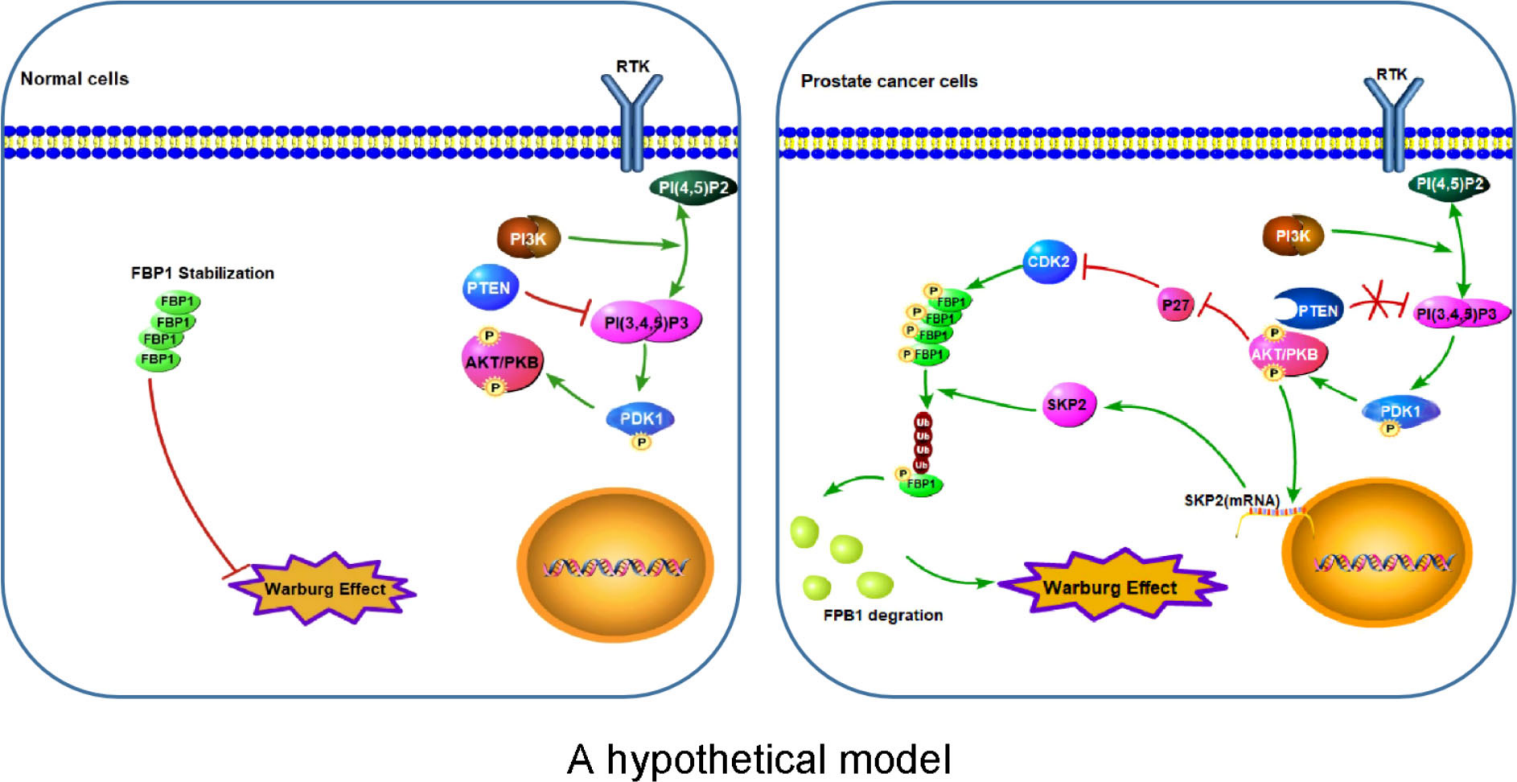
A hypothetical model Left: Stabilization of FBP1 protein prevents over-activation of the Warburg effect in normal cells. Right: Aberrant activation of the PI3K/AKT pathway due to deregulation of signaling such as loss of PTEN leads to CDK-dependent phosphorylation and ubiquitination and degradation of FBP1 mediated by SKP2 E3 ubiquitin ligase, thereby leading to abnormal activation of the Warburg effect and cell growth of cancer such as PCa. P letter with circle represents protein phosphorylation.

The major finding of the current study is the identification of a previously unrecognized molecular mechanism that promotes Warburg effect and cancer progression. FBP1, a recognized tumor suppressor, is known to inhibit cancer development *via* inhibition of aerobic glycolysis and suppression of the Warburg effect in different cancer types (4, 10, 43). In this study, we have revealed that PTEN plays a key function in regulating FBP1 expression and tumor progression through FBP1 phosphorylation-dependent ubiquitin degradation in PCa, thus defining a new role of PTEN in metabolism and tumor progression. We have found that the loss of PTEN promotes the degradation of FBP1 protein by activating the PI3K/AKT pathway *via* two mechanisms. On the one hand, activated AKT down-regulates p27, thus increasing the activity of its substrate proteins such as CDKs and promoting the CDK-dependent phosphorylation of FBP1. On the other hand, activated AKT upregulates *SKP2* mRNA and then increases the expression of SKP2 protein, which can ubiquitinate CDK-phosphorylated FBP1, and lead to CDK phosphorylation-dependent degradation of FBP1 (**Figure 7**). Our observation further support the notion that mutation in the CDK-phosphorylation site on FBP1 can hinder the degradation of FBP1 and increases FBP1 expression, thereby restraining Warburg effect and PCa growth. These findings not only provide an mechanistic explanation for the observed PTEN loss-induced downregulation of FBP1 protein in PCa, but also provide mechanistic insight into the tumor growth augmented by the Warburg effect.

FBP1 is the rate-limiting enzyme in the process of gluconeogenesis, which converts fructose-1,6-biphosphate to fructose-6-phophate and inorganic phosphate (44). Previous studies have shown that FBP1 is frequently downregulated in a variety of solid tumors (4, 10, 45). Its down-regulation mechanism may be related to DNA hypermethylation of gene promoter or loss of the copy number of the gene (4, 46), histone deacetylation caused by deregulation of HDACs (11) or degradation of FBP1 in tumor cells caused by the E3 ligase TRIM28. It is important that FBP1 downregulation is tightly related to the Warburg effect, which is a key metabolic feature of many cancers. In this study, we have demonstrated that FBP1 protein is destructed after phosphorylation in PTEN-deficient PCa. Consistent with the finding that FBP1 is often downregulated in human cancers, we provide evidence that loss of FBP1 contributes to tumor growth through the Warburg effect in PTEN-deficient PCa. Thus, our findings define a new role of FBP1 in regulating cancer progression.

PTEN plays an important role in cancer development and progression. Previous studies have reported that loss of PTEN plays a role in tumor metabolism. In our study, we have demonstrated that PTEN loss leads to phosphorylation of FBP1 at the CDK site and promotes its degradation. This effect is attenuated by mutation of CDK phosphorylation site on FBP1. Our findings reveal PTEN as an upstream protector of FBP1 and uncover a new mechanism by which PTEN regulates metabolism in cancer. The significance of these findings is further accentuated by our observations that expression of PTEN and FBP1 is positively corrected in multiple PCa cell lines and a PCa mouse model.

In summary, we have discovered a new role of PTEN in antagonizing Warburg effect by regulating expression of FBP1 and its effect on cell metabolism and tumor growth. Mechanistically, we show that PTEN inhibits phosphorylation-dependent ubiquitination-mediated degradation of FBP1. This finding highlights the importance of FBP1 protein destruction in augmented Warburg effect and growth of PTEN-deficient PCa. Our findings also suggest that targeting PTEN loss-induced FBP1 protein degradation could be a viable strategy for effective treatment of cancers such as PCa with aberrant activation of PI3K/AKT.

## Data availability statement

The original contributions presented in the study are included in the article/**Supplementary Material**. Further inquiries can be directed to the corresponding authors.

## Ethics statement

All animal study was approved by the Mayo Clinic Institutional Animal Care and Use Committee (IACUC).

## Author contributions

HH conceived the study. CS, JZ, XL, ML, ZK, JY, and JC performed experiments and collected and analyzed the data. HH and DW supervised the study. CS, JZ, ML and HH wrote the manuscript. All authors contributed to the article and approved the submitted version. CS, JZ and XL have contributed equally to this work.

## Acknowledgments

We thank Lan Shen for the technical assistance. The work was supported by funding from the Mayo Clinic Foundation (to HH).

## Conflict of interest

The authors declare that the research was conducted in the absence of any commercial or financial relationships that could be construed as a potential conflict of interest.

## Publisher’s note

All claims expressed in this article are solely those of the authors and do not necessarily represent those of their affiliated organizations, or those of the publisher, the editors and the reviewers. Any product that may be evaluated in this article, or claim that may be made by its manufacturer, is not guaranteed or endorsed by the publisher.
