# Supplementary material for: PTEN loss promotes Warburg effect and prostate cancer cell growth by inducing FBP1 degradation
Source: Front Oncol. 2022 Sep 27;12:911466. doi: 10.3389/fonc.2022.911466 (PMC9552847; doi:10.3389/fonc.2022.911466)
Supplement: Supplementary file 1 [file Table_1.docx]

Supplementary Table 1. Sequences of primers for RT-qPCR and PCR

| For RT-qPCR (Human) |  |  |
| --- | --- | --- |
| Gene | Forward | Reverse |
| GAPDH | ACCCAGAAGACTGTGGATGG | TTCAGCTCAGGGATGACCTT |
| FBP1 | ACATCGATTGCCTTGTGTCC | CCACCAAAATGAACTCCCCG |
| PTEN | TTGGCGGTGTCATAATGTCT | GCAGAAAGACTTGAAGGCGTA |
| SKP2 | GTGGTATCGCCTAGCGTCTG | GAGACAGTATGCCGTGGAGG |

| For PCR (Mus musculus) |  |  |
| --- | --- | --- |
| Gene | Forward | Reverse |
| Pten | ATGACAGCCATCATCAAAGAGATC | TCAGACTTTTGTAATTTGTGAATGCT |
