# Supplementary material for: PTEN loss promotes Warburg effect and prostate cancer cell growth by inducing FBP1 degradation
Source: Front Oncol. 2022 Sep 27;12:911466. doi: 10.3389/fonc.2022.911466 (PMC9552847; doi:10.3389/fonc.2022.911466)
Supplement: Supplementary file 2 [file Table_2.docx]

Supplementary Table 2. Sequences of shRNAs

| Gene | Sequence |
| --- | --- |
| shPTEN-1 | CCGGAGGCGCTATGTGTATTATTATCTCGAGATAATAATACACATAGCGCCTTTTTT |
| shPTEN-2 | CCGGCTAGAACTTATCAAACCCTTTCTCGAGAAAGGGTTTGATAAGTTCTAGTTTTT |
| shSKP2-1 | CCGGGCCTAAGCTAAATCGAGAGAACTCGAGTTCTCTCGATTTAGCTTAGGCTTTTT |
| shSKP2-2 | CCGGCCACGATCATTTATGGACCAACTCGAGTTGGTCCATAAATGATCGTGGTTTTT |
